# Gas Vesicle–Blood Interactions Enhance Ultrasound Imaging Contrast

**DOI:** 10.1021/acs.nanolett.3c02780

**Published:** 2023-11-20

**Authors:** Bill Ling, Jeong Hoon Ko, Benjamin Stordy, Yuwei Zhang, Tighe F. Didden, Dina Malounda, Margaret B. Swift, Warren C. W. Chan, Mikhail G. Shapiro

**Affiliations:** † Division of Chemistry and Chemical Engineering, California Institute of Technology, Pasadena, California 91125, United States; ‡ Institute of Biomedical Engineering, University of Toronto, Toronto, ON M5S 3G9, Canada; § Terrence Donnelly Centre for Cellular & Biomolecular Research, University of Toronto, Toronto, ON M5S 3E1, Canada; ∥ Department of Chemistry, University of Toronto, Toronto, ON M5S 3H6, Canada; ⊥ Division of Engineering and Applied Science, California Institute of Technology, Pasadena, California 91125, United States; # Howard Hughes Medical Institute, California Institute of Technology, Pasadena, California 91125, United States

**Keywords:** gas vesicles, ultrasound imaging, blood, protein corona, surface engineering

## Abstract

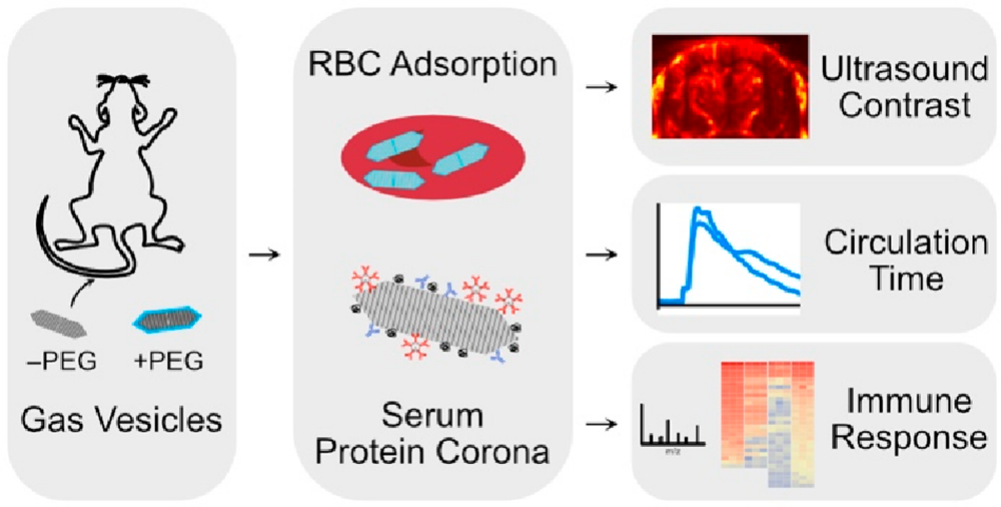

Gas vesicles (GVs) are genetically encoded, air-filled protein nanostructures of broad interest for biomedical research and clinical applications, acting as imaging and therapeutic agents for ultrasound, magnetic resonance, and optical techniques. However, the biomedical applications of GVs as systemically injectable nanomaterials have been hindered by a lack of understanding of GVs’ interactions with blood components, which can significantly impact *in vivo* behavior. Here, we investigate the dynamics of GVs in the bloodstream using a combination of ultrasound and optical imaging, surface functionalization, flow cytometry, and mass spectrometry. We find that erythrocytes and serum proteins bind to GVs and shape their acoustic response, circulation time, and immunogenicity. We show that by modifying the GV surface we can alter these interactions and thereby modify GVs’ *in vivo* performance. These results provide critical insights for the development of GVs as agents for nanomedicine.

Nanomaterials are becoming increasingly important for biomedical applications such as drug delivery, medical imaging, and diagnostics.^1^ In these contexts, nanoparticle behavior is significantly impacted by the cells and proteins encountered within the bloodstream. Serum proteins rapidly adsorb to nanoparticle surfaces, forming a protein corona that alters their physicochemical properties and recognition by the body.^2−5^ The corona’s composition can predict factors such as pharmacokinetics, biodistribution, toxicity, and cellular uptake.^6−8^ Modification strategies often involve covering the particle surface with hydrophilic polymers such as poly(ethylene glycol) (PEG) and other ligands.^9^ Additionally, some nanomaterials bind to erythrocytes (RBCs), affecting imaging contrast,^10^ biodistribution,^11^ and circulation time.^12^

Gas vesicles (GVs) are an emerging nanomaterial with great potential as agents for imaging and therapy.^13^ These air-filled protein nanostructures are naturally produced by certain aquatic microbes for buoyancy regulation.^14^ GVs comprise a 2 nm thick protein shell that excludes liquid water but permits the dynamic exchange of gas, forming a thermodynamically stable pocket of air with nanoscale dimensions.^14^ Acoustic waves are strongly scattered at this air–water interface, enabling GVs to produce robust ultrasound contrast when injected into the body^15−17^ or expressed in engineered cells.^18,19^ Furthermore, they are resilient to repeated insonation,^15^ easily tailored to target molecular markers^20−22^ or respond to biological functions,^23,24^ and have growing applications in therapeutic ultrasound,^25,26^ optical imaging,^27,28^ and magnetic resonance imaging.^29,30^ To effectively incorporate these capabilities into an injectable agent, a deeper understanding of *in vivo* GV behavior is needed.

In this study, we investigate GV interactions with RBCs and serum proteins, develop surface functionalization techniques to modulate these interactions, and evaluate the downstream effects on the acoustic response, circulation time, and immunogenicity. We characterize GVs’ protein corona and identify molecular pathways governing their *in vivo* fate. This analysis offers valuable insights for the ongoing development and optimization of injectable nanoparticles and GV-based agents.

We began by studying the behavior of GVs after intravenous (IV) administration. We visualized circulating GVs with ultrafast power Doppler ultrasound imaging, leveraging their ability to enhance blood flow contrast.^16^ Targeting a single coronal plane in the mouse brain, we acquired images at a 15.625 MHz center frequency and a 0.25 Hz frame rate (Figure 1A). After a 5 min baseline, we injected 100 μL of 5.7 nM GVs purified from *Anabaena flos-aquae*(31) (Ana) and monitored the ensuing changes in hemodynamic signal. In healthy BALB/c mice, contrast reached an initial peak within 1 min, followed by a larger peak 3.5 min later, and then returned to baseline over approximately 30 min as GVs were cleared by the liver^24^ (Figure 1B). Intensities at the first peak were consistent across trials but varied significantly at the second peak (Figure 1C).

**Figure 1:**
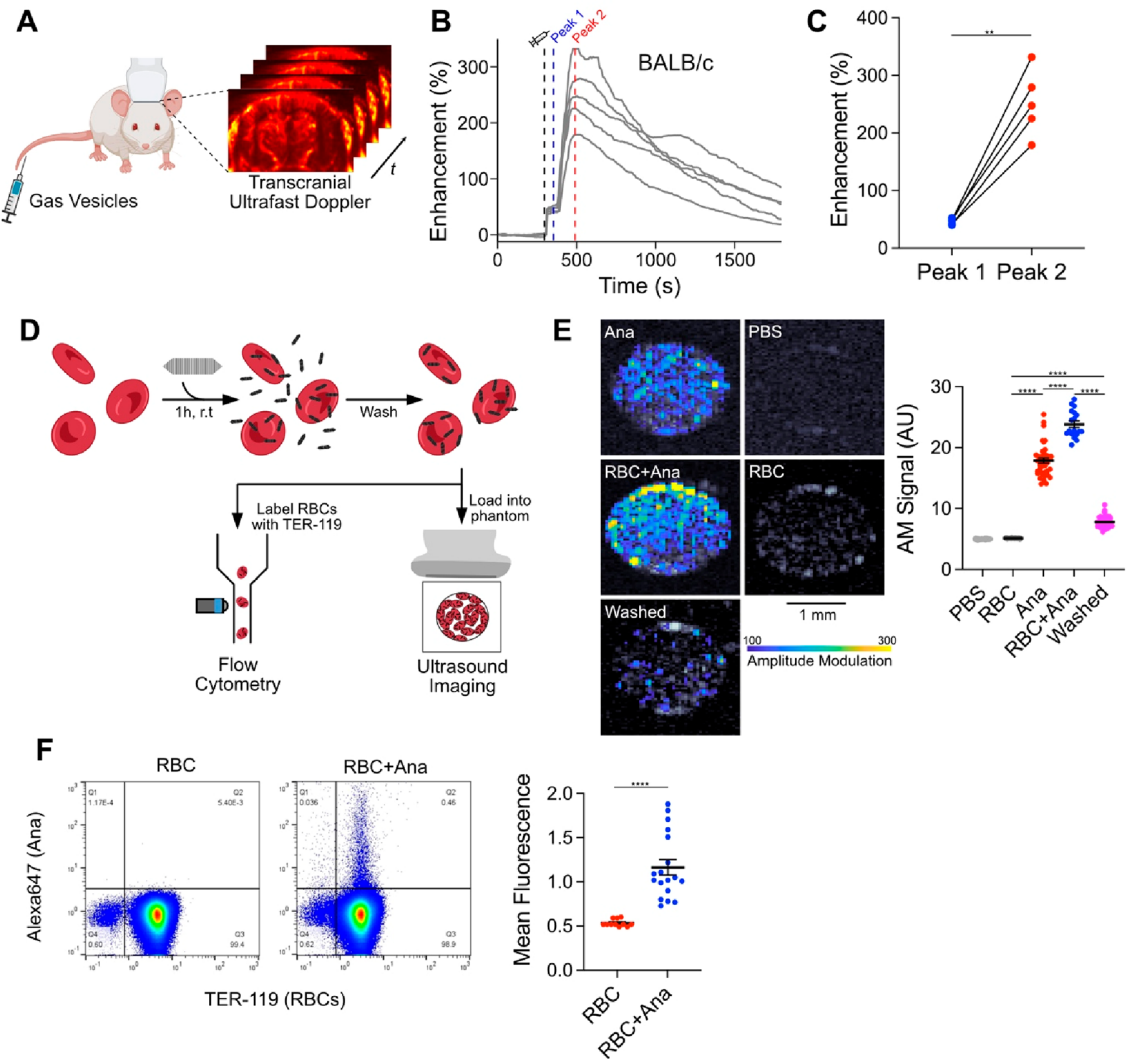
GV adsorption to RBCs contributes to a second peak of the hemodynamic ultrasound contrast. (A) Diagram of the *in vivo* imaging setup. Intravascular dynamics of IV injected GVs were visualized by transcranial ultrafast power Doppler imaging of the brain. (B) Time courses of Doppler signal enhancement in immunocompetent BALB/c mice following injection of 100 μL of 5.7 nM GVs. *N* = 5. Dashed gray line, time of injection (300 s); dashed blue line, peak 1 (350 s); dashed red line, peak 2 (480 s). (C) Signal enhancement at the indicated peaks in time courses from panel B. Points from the same trial are connected. *N* = 5. Paired *t* test, (**p < 0.01). (D) Diagram of the RBC binding assay. Ultrasound imaging: RBCs were incubated with GVs modified to produce nonlinear signal, washed by centrifugation, and loaded into an agarose phantom for nonlinear AM imaging. Flow cytometry: RBCs were incubated with fluorescently labeled GVs, washed by centrifugation, stained with anti-TER-119, and analyzed by flow cytometry. (E) Acoustic detection of adsorbed GVs. Left: representative ultrasound images of RBCs mixed with GVs. AM signal is overlaid on a B-mode image to show sample outlines. Scale bars: 1 mm. Right: mean AM signal intensity within each well. *N* = 18–60. Error bars: ±SEM. Welch’s *t* test (****p < 0.0001). (F) Flow cytometric detection of GVs adsorbed to RBCs. Left: representative scatter plots of washed RBCs, gated for single cells. The gating strategy is shown in Figure S5. Right: mean fluorescence of TER-119+ cell population. RBC, *N* = 11; RBC+Ana, *N* = 18. Error bars: ±SEM. Welch’s *t* test (****p < 0.0001).

We next investigated the cause of this dual-peak phenomenon. We hypothesized that the first peak was due to dispersion of free-floating GVs throughout the bloodstream, as its onset time matched the vascular distribution kinetics of other nanoparticle and small molecule contrast agents.^32,33^ Furthermore, the intensity of the first peak correlated linearly with injected dose, suggesting that it is directly governed by GV concentrations in the blood (Figure S1). In comparison, the intensity of the second peak appeared to plateau at higher doses, suggesting a binding interaction.

We hypothesized that the second peak arose from an increase in acoustic backscatter due to GV clustering.^15^ We observed similar contrast enhancement dynamics in immunocompetent BALB/c (Figure 1B) and immunocompromised NSG mice (Figure S2), which lack both B cells and T cells, and therefore suspected an antibody-independent mechanism such as adsorption to RBCs, as previously seen with nanobubbles.^10^ To evaluate this concept, we calculated the theoretical scattering cross section^34^ of RBC-GV complexes. Modeled as uniform spheres with volume-weighted physical properties, the scattering cross section increased with the number of adsorbed GVs and was greater than that of dispersed particles (Figure S3).

To quantify adsorption, we exposed purified mouse RBCs in phosphate-buffered saline (PBS) to GVs that were modified to produce nonlinear ultrasound contrast.^22^ The RBCs were maintained at approximately 4% hematocrit (10% of whole blood) to facilitate uniform mixing, while the GVs were at concentrations approximating *in vivo* conditions following vascular dispersion (0.2 nM). After 1 h, we loaded the samples into an imaging phantom and detected GVs specifically with an amplitude modulation (AM) pulse sequence^35^ (Figure 1D). Signal intensities for RBC and GV controls were 5.12 ± 0.03 and 17.89 ± 0.42 AU, respectively, and increased to 23.85 ± 0.53 AU after mixing. After centrifugation to remove unbound GVs, 7.74 ± 0.11 AU (21% of GV control) was retained (Figure 1E). Notably, GV contrast was less impacted by RBC addition in the presence of serum, suggesting a reduction in the extent of adsorption (Figure S4).

We validated these results by exposing RBCs in 1% bovine serum albumin (BSA) in PBS to GVs labeled with fluorescent dye. After 1 h, we washed the cells thoroughly to remove loosely bound GVs and analyzed them by flow cytometry (Figures 1D and S5). The mean fluorescence of the population doubled from 0.53 ± 0.01 to 1.17 ± 0.09, with 0.5% of the RBCs showing significant binding (Figure 1F).

Taken together, our data suggest that increased acoustic backscatter from GV adsorption to RBCs could contribute to the delayed wave of hemodynamic contrast. Consistent with previous studies on nanoparticle adhesion to RBCs,^36−38^ binding saturated at higher doses (Figure S1) and was diminished in the presence of competing proteins such as serum (Figure S4). The ability of serum proteins to inhibit binding suggests that this process occurs primarily through nonspecific adsorption, though specific biomolecular interactions cannot be excluded. In the body, it may operate in concert with other mechanisms, such as serum-induced aggregation, to influence acoustic contrast—a phenomenon that we examine below.

To minimize RBC adsorption, we engineered GVs coated with methoxypoly(ethylene glycol) (mPEG), a widely used polymer for nanoparticle passivation.^9^ We functionalized lysines on the GV surface with alkyne groups and attached 10 kDa mPEG-azides through a copper-catalyzed azide–alkyne cycloaddition (CuAAC) (Figure 2A). This size of mPEG has previously shown efficacy with GVs^39^ and is expected to graft in a brush conformation based on mass spectrometric estimates of alkyne density (Figure S6). We will refer to unmodified GVs as Ana and to functionalized GVs as Ana-PEG. Consistent with the addition of a PEG layer, dynamic light scattering (DLS) showed an increase in hydrodynamic diameter from 240 ± 4 to 370 ± 12 nm (Figure 2B), while the zeta-potential neutralized from −56 ± 1.5 to −5 ± 0.3 mV (Figure 2C). We next performed pressurized absorbance spectroscopy, which tracks optical density under increasing hydrostatic pressure to determine the threshold at which GVs collapse, providing a convenient measure of structural integrity.^15,31^ Ana and Ana-PEG collapsed at 600 and 450 kPa, respectively, suggesting that attachment of mPEG mildly destabilized the GV shell but that most of its strength was intact (Figure 2D). Incubation with mPEG or CuAAC reagents separately had no effect (Figure S7), while direct functionalization with NHS-PEG severely compromised shell stability and failed to shield surface charge (Figure S8). B-mode ultrasound contrast from both GV types was equivalent (Figure 2E).

**Figure 2:**
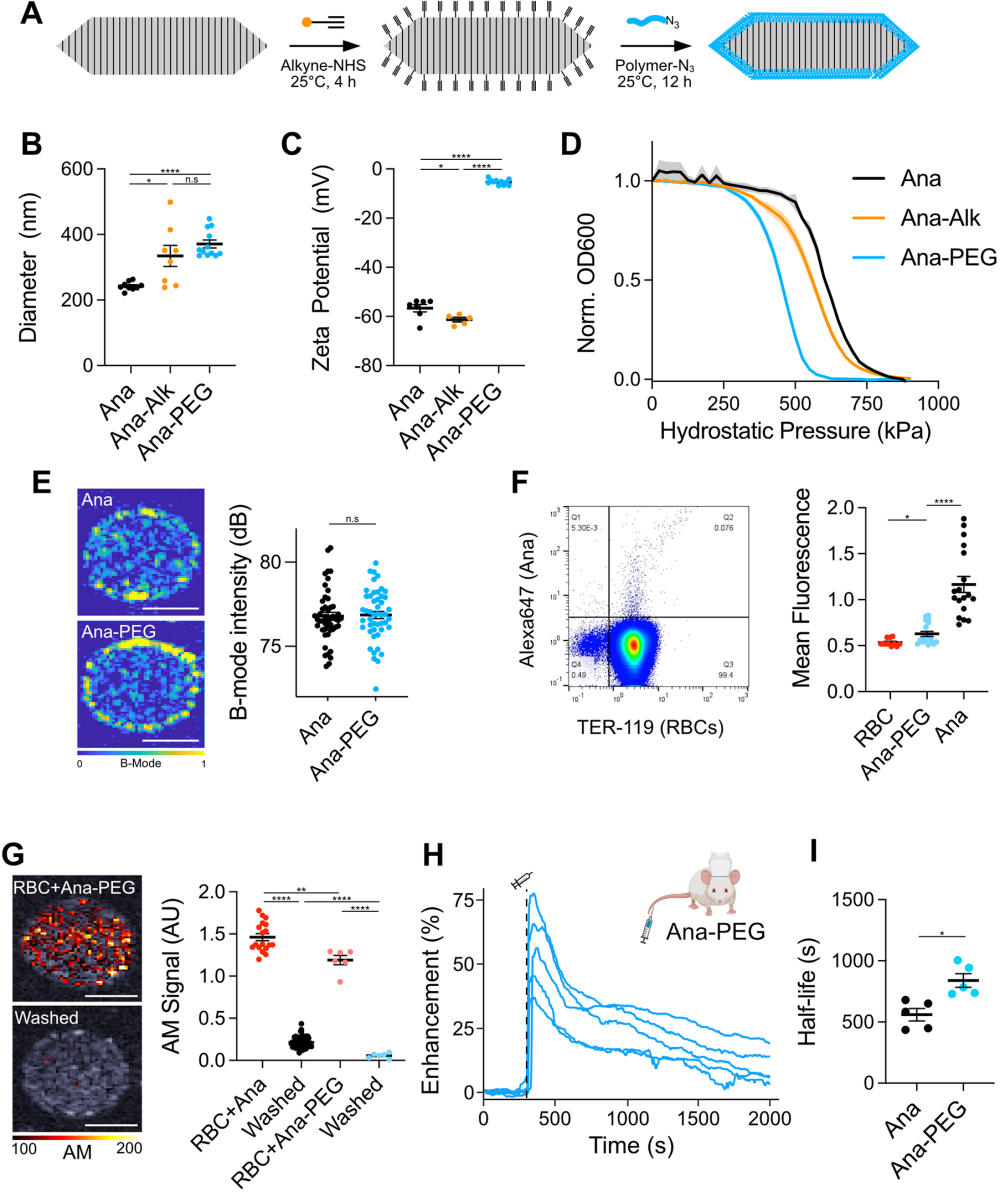
Surface passivation reduces RBC binding and extends the circulation time. (A) Reaction scheme for GV functionalization. Alkynes were conjugated to lysines on the GV surface, and polymers were attached through a CuAAC reaction. (B) DLS measurements of the hydrodynamic diameter. *N* = 8–12. Error bars: ±SEM. Welch’s *t* test (*p < 0.05; ****p < 0.0001; n.s, *p* ≥ 0.05). (C) Zeta-potential measurements of engineered GVs. *N* = 5–11. Error bars: ±SEM. Welch’s *t* test (*p < 0.05; ****p < 0.0001). (D) Normalized optical density at 600 nm as a function of hydrostatic pressure. *N* = 4. Thick lines: mean; shaded areas: ±SEM (E) Left: representative B-mode images of Ana and Ana-PEG embedded in an agarose phantom. Right: mean B-mode signal intensities were within each well. *N* = 48. Error bars: ±SEM. Welch’s *t* test (n.s., *p* ≥ 0.05). (F) Flow cytometric detection of fluorescently labeled Ana-PEG adsorbed to RBCs. Left: representative dot plot of washed RBCs, gated for single cells. RBCs are stained with anti-TER-119. Right: mean fluorescence of TER-119+ cell population. *N* = 18. Ana and RBC-only controls from Figure 1F are shown as a reference. Error bars: ±SEM. Welch’s *t* test (*p < 0.05; ****p < 0.0001). (G) Acoustic detection of Ana-PEG modified to produce nonlinear contrast. Left: representative ultrasound images of RBCs were mixed with Ana-PEG. The AM signal is overlaid on a B-mode image. Right: AM signal intensities were normalized to their respective GV-only samples. *N* = 6. Normalized data from Figure 1E are shown for comparison. Error bars: ±SEM. Welch’s *t* test (**p < 0.01; ****p < 0.0001). (H) Time courses of ultrafast power Doppler signal enhancement following injection of Ana-PEG into BALB/c mice. *N* = 5. Dashed line is the time of injection (300 s). (I) Half-life of GV-induced signal enhancement calculated by fitting time courses in Figure 1B (Ana) and Figure 2H (Ana-PEG) to an exponential decay function. Error bars: ±SEM. Welch’s *t* test (*p < 0.05).

Next, we evaluated the effectiveness of this coating at reducing the RBC adsorption. As before, we mixed purified mouse RBCs with fluorescently labeled Ana-PEG for 1 h, removed loosely bound GVs by centrifugation, and analyzed the cells by flow cytometry (Figure 1D). Less than 0.1% of cells exhibited significant binding, with mean fluorescence of the population only increasing to 0.63, compared to 1.17 for Ana (Figure 2F). Likewise, less than 5% of the ultrasound signal was retained after washing away unbound Ana-PEG compared to 21% with Ana (Figure 2G).

Having confirmed the reduced adsorption of Ana-PEG to RBCs, we assessed their *in vivo* behavior. Following IV injection of 100 μL of 5.7 nM Ana-PEG into healthy BALB/c mice, hemodynamic contrast reached a maximum within 1 min before returning to baseline monotonically (Figure 2H). The timing and magnitude of enhancement at this peak were consistent with the first peak observed after Ana injection (Figure 1B), supporting our hypothesis that this initial peak resulted from vascular distribution. Unlike in the Ana time course, however, a second peak of contrast enhancement was not observed. Fitting these time courses to an exponential decay model, we found that PEGylation increased apparent circulation half-life from 560 ± 51 to 840 ± 56 s (Figure 2I).

GV aggregation is an alternative mechanism to increasing acoustic backscatter^15^ which cannot be excluded by the results presented thus far. Because of their highly charged surfaces (Figure 2C), GV aggregation is unlikely to occur spontaneously^40^ and would most likely be facilitated by a component within serum. To test this idea, we incubated Ana and Ana-PEG in 80% serum from naïve BALB/c, NSG, and outbred non-Swiss mice for 1 h at 37 °C and measured flotation, a reliable indicator of clustering,^15^ by comparing optical densities at the surface and in the subnatant (Figure 3A). We included NSG mice due to their lack of antibodies, while the genetic heterogeneity of outbred mice increases the likelihood of forming native antibodies^41^ against GVs. Prior to incubation, the optical density ratio was 1.1 for both GV types and increased only slightly in BALB/c and NSG serum (without statistical significance; Figure 3B). Upon exposure to outbred serum, Ana formed a distinct buoyant layer (ratio 1.9 ± 0.05), while Ana-PEG showed a less pronounced increase (ratio 1.3 ± 0.04). Preincubation transmission electron microscopy (TEM) images contained only discrete particles (Figures 3C and S9). Exposure to outbred serum caused Ana to assemble into multi-GV bundles, whereas Ana-PEG formed occasional small clusters but remained mostly dispersed.

**Figure 3:**
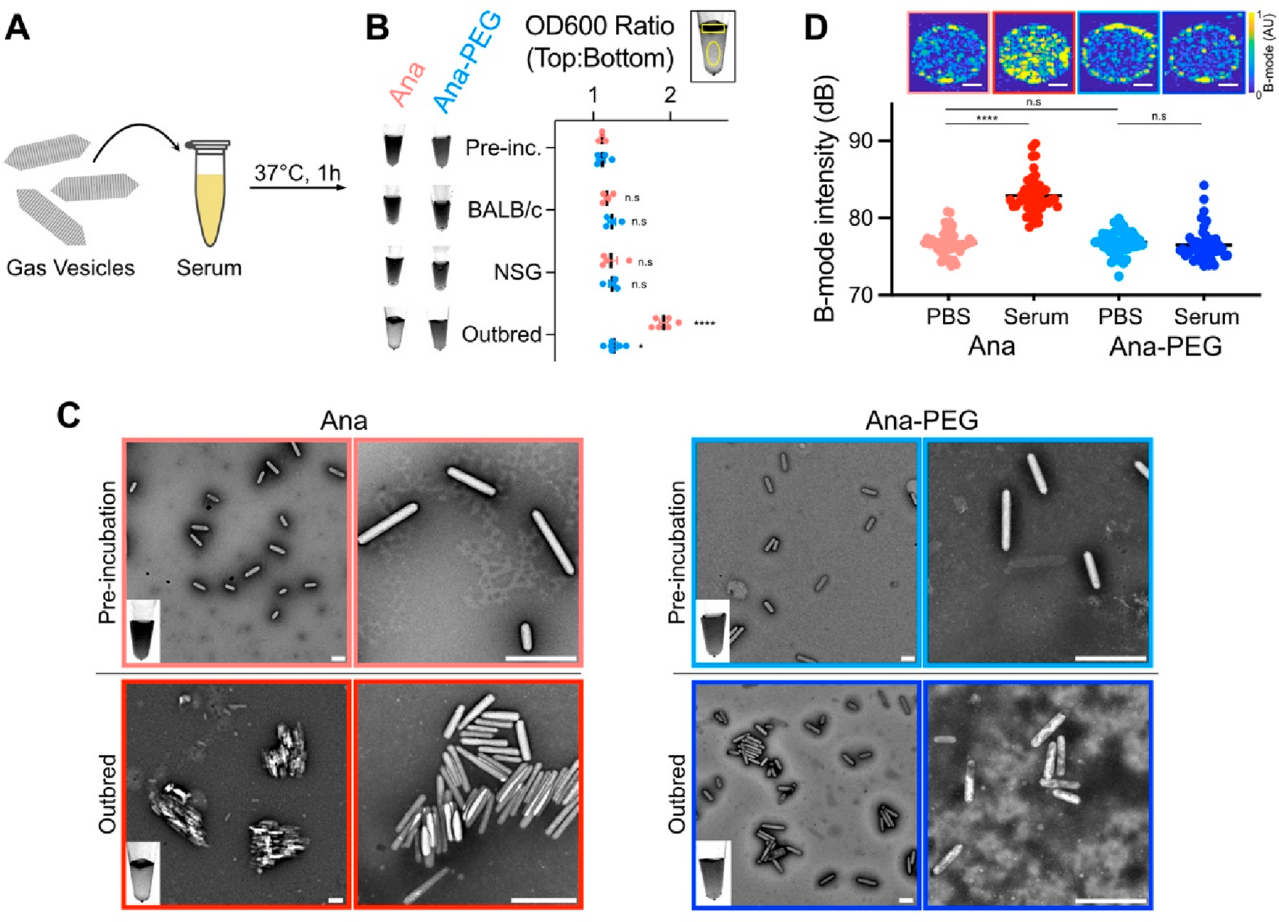
Serum exposure can cause GVs to aggregate. (A) Diagram of the serum incubation assay. GVs (0.23 nM) were incubated in 80% mouse serum for 1 h at 37 °C. (B) Optical detection of GV flotation. Left: representative transillumination images of serum-incubated GVs. Right: ratio of optical densities in manually drawn ROIs at the surface and in the subnatant of each sample. Representative ROIs are shown above the plot. Error bars: ±SEM. Welch’s *t* test compared to preincubation samples (*p < 0.05; ****p < 0.0001; n.s, *p* ≥ 0.05). (C) Representative TEM images of GVs before and after exposure to serum from outbred mice. Inset shows a transillumination image of the corresponding sample. Scale bars: 500 nm. Additional images are shown in Figure S9. (D) Ultrasound imaging of GVs following incubation in PBS or outbred mouse serum. Top: representative B-mode images. Scale bars: 1 mm. Bottom: mean signal intensity within each well. *N* = 48. Error bars: ±SEM. Welch’s *t* test (****p < 0.0001; n.s, *p* ≥ 0.05).

To compare acoustic responses, we embedded GVs treated with either PBS or outbred serum into an imaging phantom and acquired ultrasound images using a B-mode pulse sequence (Figure 3D). Signal intensity from Ana increased by 6.13 dB in outbred serum relative to PBS, while the contrast from Ana-PEG remained relatively unchanged. In BALB/c serum, the signal from Ana increased by 2.26 dB, consistent with a lower degree of aggregation compared to outbred serum (Figure S10). Taken together, these results demonstrate that antibodies and other serum factors can aggregate GVs, triggering a substantial increase in the acoustic contrast.

We next investigated the impact of elicited antibodies on GV dynamics *in vivo* by administering multiple GV injections to the same animals. We injected BALB/c mice with an initial dose of 100 μL of 5.7 nM GVs and did so again 1 or 4 weeks later (Figure 4A). Ana injections resulted in peak enhancements of approximately 280% at all three time points (Figure 4B,C), with apparent circulation half-life decreasing from 560 ± 51 s to 290 ± 11 s and 410 ± 36 s at weeks 1 and 4, respectively (Figure 4D). Repeated Ana injections were well-tolerated with no health anomalies observed by veterinary assessment.

**Figure 4:**
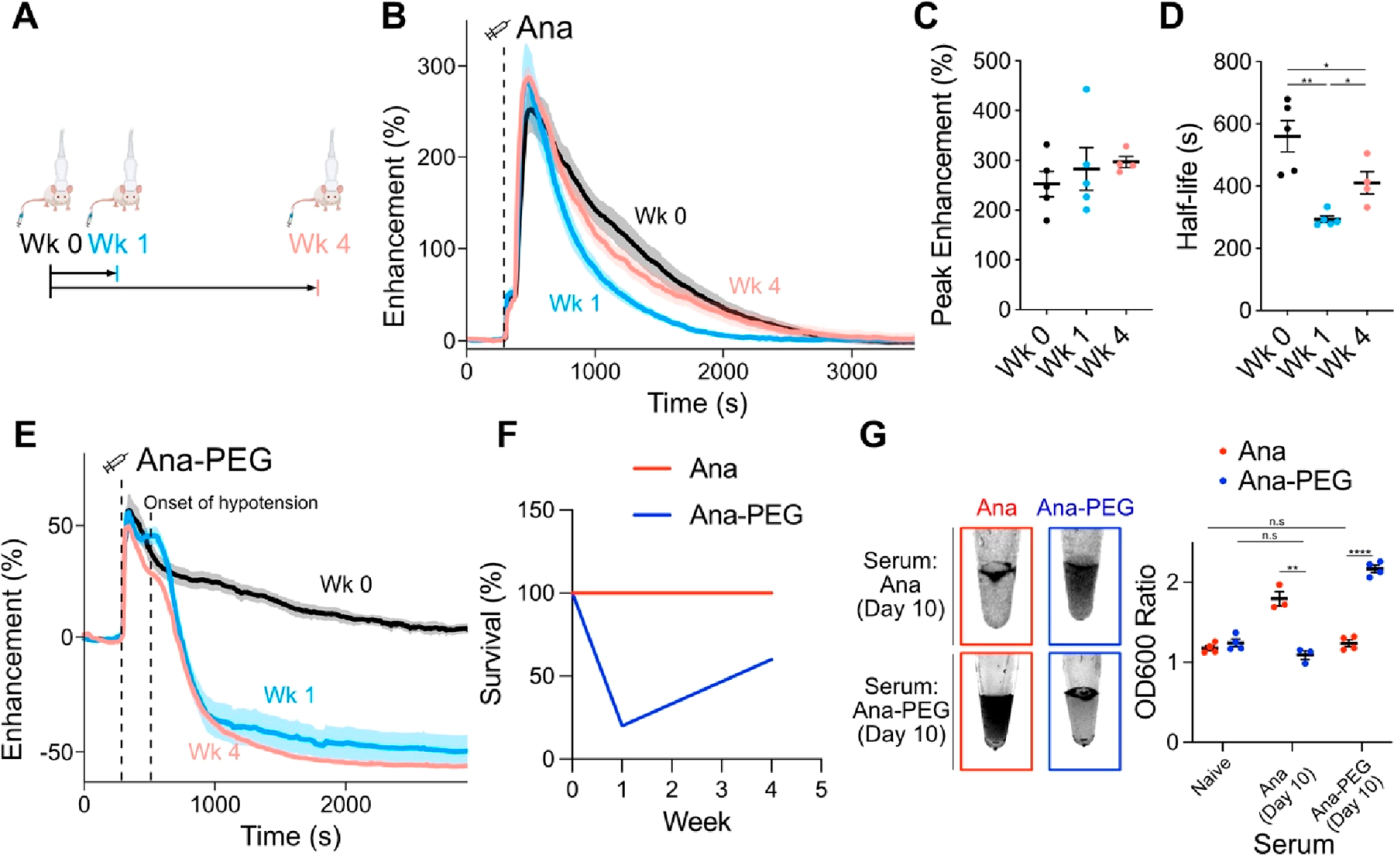
Antibody reactions to GVs. (A) Timeline of GV injections. Immunocompetent mice were injected with two doses of 100 μL of 5.7 nM Ana or Ana-PEG, separated by either 1 or 4 weeks. Ultrafast Doppler imaging was performed at each injection. (B) Time courses of hemodynamic signal enhancement following administration of Ana. Thick lines: mean; shaded area: ±SEM; dashed line: time of injection (300 s). *N* = 4–5. (C) Peak enhancement of time courses in panel B. Error bars: ±SEM. (D) Circulation half-life calculated by fitting time courses in panel B to an exponential decay function. Error bars: ±SEM. Welch’s *t* test (*p < 0.05; **p < 0.01). (E) Time courses of hemodynamic signal enhancement following administration of Ana-PEG. Severe hypotension occurred within 5 min of injection, resulting in a sharp drop in the hemodynamic signal. Thick lines: mean; shaded area: ±SEM; dashed lines: time of injection (300 s) or onset of hypotension (500 s). *N* = 4–5. (F) Survival rate following GV administration. All mice dosed with Ana recovered after both injections. Several mice dosed with Ana-PEG did not recover after the second injection. *N* = 5 at each time point. (G) GV aggregation in the presence of anti-GV antibodies. Left: representative transillumination images of GVs incubated in serum prepared from mice 10 days postimmunization. Right: ratio of optical density at the surface relative to that of the subnatant. Values from incubation in naïve BALB/c serum (Figure 3B) are included for comparison. *N* = 3–4. Error bars: ±SEM. Welch’s *t* test (**p < 0.01; ****p < 0.0001; n.s, *p* ≥ 0.05).

Ana-PEG produced peak enhancements of approximately 50% at all three time points (Figures 4E and S11). However, acute hypotension occurred unexpectedly several minutes after the second dose of Ana-PEG, resulting in a sharp reduction in the hemodynamic contrast (Figure 4E). Only 20% of mice recovered from hypotension at week 1 and 60% at week 4 (Figure 4F). Based on similar responses to other PEGylated materials,^42,43^ we hypothesized that this reaction is triggered by anti-PEG antibodies.

To further investigate antibody interactions, we exposed GVs to serum obtained from mice 10 days after the initial GV injection (Figure 4G). In the presence of Ana-PEG antiserum, Ana-PEG aggregated and formed a buoyant layer within 30 min, while Ana remained in suspension. Conversely, Ana rapidly aggregated upon exposure to Ana antiserum, while Ana-PEG remained in suspension. This pattern is consistent with a clear distinction in antibody selectivity and minimal cross-reactivity for the different GV surfaces. Substituting mPEG with a 16 kDa zwitterionic polymer, which is being explored as a less immunogenic PEG alternative,^44,45^ did not alleviate coating-induced anaphylaxis (Figure S12).

Taken together, our data suggest that RBC adsorption is likely the primary contributor to GV-enhanced hemodynamic contrast, and serum components mediate particle clearance and elicit immune responses. This conclusion is supported by the comparable Doppler signal dynamics observed in antibody-deficient NSG (Figure S2), naïve BALB/c (Figure 1B), and GV-exposed BALB/c mice (Figure 4B). Furthermore, peak enhancement was not impacted by the presence of serum factors capable of causing significant GV aggregation (Figure 4C,G). Instead, these factors triggered an acceleration in GV clearance or, in the case of Ana-PEG, an infusion reaction (Figure 4D,F).

To identify the serum components influencing GV behavior, we characterized the protein coronas associated with Ana and Ana-PEG. We incubated both GV types in serum from outbred mice for 1 h at 37 °C, as it offers a more diverse representation of serum components and enhances generalizability for translational applications.^46^ After removing unbound proteins by centrifugation, we digested bound proteins with trypsin and quantified peptides by liquid chromatography tandem mass spectrometry (LC-MS/MS) (Figure 5A; see the Supporting Information for a full data set). Levels of detected proteins showed moderate correlation between serum-exposed GV types (*R* = 0.77) but less so with the background serum (Ana R = 0.58, Ana-PEG R = 0.45) (Figure S13), indicating that Ana and Ana-PEG selectively enriched for similar proteins through a process that cannot be explained by serum concentration alone. We detected comparable amounts of the GV structural protein GvpC across all samples, indicating similar GV loading, as well as minor quantities of GvpV, GvpN, and other cyanobacterial proteins (Figure S14).

**Figure 5:**
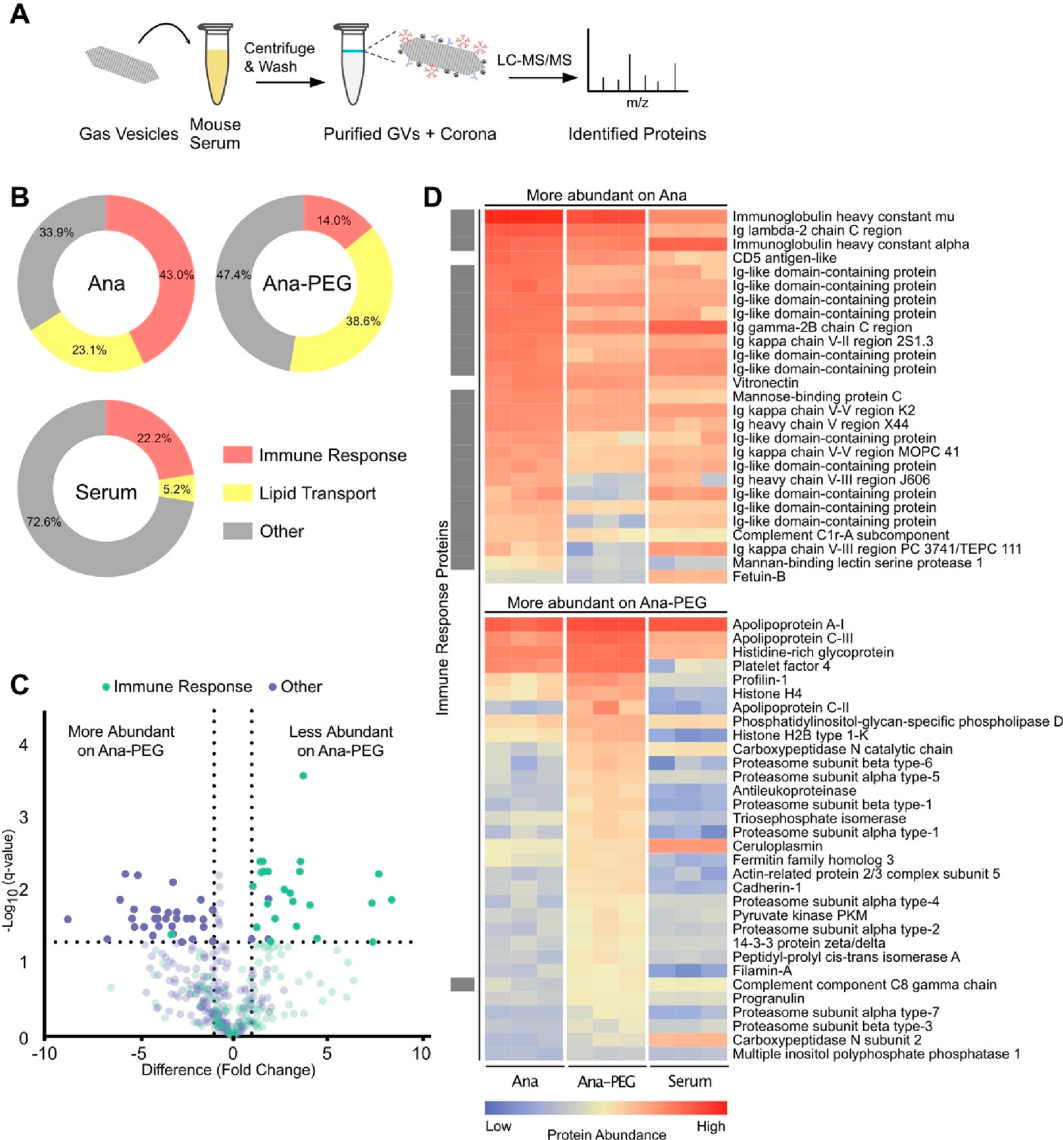
Characterization of the GV protein corona. (A) Schematic of the corona analysis protocol. GVs were incubated in outbred mouse serum for 1 h at 37 °C, purified by centrifugation, and processed for LC-MS/MS. (B) Donut charts of relative abundances of immune response and lipid transport proteins, as classified by gene ontology. (C) Volcano plot of protein abundance on Ana compared to Ana-PEG. Abundances were compared by multiple unpaired *t* test analysis using the false discovery rate method of Benjamini and Hochberg. Proteins with a false discovery rate of 5% and log_2_ fold change greater than 1 were deemed differentially abundant. Proteins that were not differentially abundant are shown translucently. (D) Heat map of differentially abundant proteins on Ana compared to Ana-PEG, shown with a mouse serum control. Immune response proteins are indicated by gray boxes on the left.

Immune response proteins—part of an immunoglobulin complex or involved in complement activation—were highly abundant in the Ana corona, constituting 43.0% of detected proteins, compared to 14.0% of the Ana-PEG corona and 22.2% of serum (Figure 5B). Of the proteins significantly more enriched on Ana than Ana-PEG, 24 of 27 were associated with immune response, including immunoglobulin A, immunoglobulin G, immunoglobulin kappa, mannose-binding protein C, and complement C1r (Figure 5C,D). Similarly, 13 of the top 25 proteins enriched on Ana relative to serum were in this group (Figure S15). In contrast, only one immune response protein was among the 32 proteins more abundant on Ana-PEG than Ana. Consistent with its role as an early response antibody,^47^ immunoglobulin M (IgM) was the most prevalent member of this group, accounting for 15% of the Ana corona (1st overall) and 4% of the Ana-PEG corona (8th overall). Given its enrichment and multivalency, IgM is likely responsible for GV aggregation in naïve serum.

Lipid transport proteins were enriched on both GV types, comprising 23.1% and 38.6% of the Ana and Ana-PEG coronas, respectively, compared to 5.2% of the serum (Figure 5B). Apolipoprotein E (ApoE) and apolipoprotein C-I (ApoC-I) were the most prominent. On Ana, they ranked second and fourth in overall abundance with 130-fold and 50-fold enrichment relative to serum, respectively (Figure S15). On Ana-PEG, they were first and second with 200-fold and 90-fold enrichment, respectively. Ana-PEG also enriched several other proteins that are typically found in low concentrations in serum, such as apolipoprotein C-II, apolipoprotein C-III, platelet factor 4, and profilin-1 (Figures 5D and S15). Neither GV type appreciably adsorbed albumin despite its high concentration in serum.

Our results demonstrate that blood components significantly influence GV behavior in the bloodstream, highlighting opportunities for optimizing GV-based diagnostic and therapeutic agents. Injected GVs adsorb to the surface of RBCs, likely through physical mechanisms such as electrostatic interactions between proteins in the GV shell and the RBC surface, resulting in a considerable enhancement of hemodynamic contrast. Additionally, GVs acquire a serum protein corona that is rich in apolipoproteins and immunoglobulins. These corona proteins can facilitate rapid clearance and amplify the immune response upon repeated exposure. Surface passivation with 10 kDa mPEG reduces RBC and protein adsorption, providing a modest extension of circulation time at the expense of diminished blood flow contrast.

GV-based diagnostic agents could benefit from strategies to modify the protein corona, which can mask the elements required for molecular detection and response. Potential techniques include genetic functionalization of the GV surface,^22^ wrapping with cell membranes,^48^ ligand conjugation to serum-equilibrated GVs,^49^ fusion of peptides to recruit specific serum proteins,^50^ and adsorption of an artificial corona.^51^ These strategies could also enable *in vitro* diagnostic applications, such as clustering-based detection in which GVs selectively enriched with specific proteins are combined with the corresponding antibodies, allowing for rapid optical and acoustic measurements via flotation and enhanced ultrasound backscatter,^15,52^ respectively. Furthermore, the GV corona can aid in proteomics by reducing the dynamic range of protein concentrations in biological fluids, facilitating detection of low-abundance components.^53^ GVs are advantageous for these applications due to their easy buoyancy-based isolation and use of structural proteins as internal concentration standards.

To maximize the translational utility of GVs, immunogenic components should be identified and eliminated. GVs do not appear to be immunotoxic, as repeated injections of Ana were well-tolerated. However, antibodies did form against GVs, leading to accelerated clearance. Notably, several residual cyanobacterial proteins remained after GV purification. Future work could study responses to urea-treated GVs lacking these proteins,^31^ identify problematic epitopes by analyzing peptides displayed on antigen-presenting cells after lysosomal processing of the GV,^54^ and redesign production and purification processes to address these challenges. Alternatively, the strong antibody response against Ana-PEG suggests a powerful adjuvanting effect by GV-associated proteins, which could be helpful for vaccine delivery^55^ and other immunomodulatory applications.

Enhancing GV binding to RBCs could potentially alleviate immunogenicity concerns by inducing peripheral tolerance,^56,57^ while also extending GV circulation time^58,59^ and improving contrast in functional ultrasound imaging.^16^ Approaches include covalent linkage to engineered RBCs^58^ or incorporation of RBC affinity ligands to enhance binding *in situ*.^59^ Future work should also examine the impact of GV adsorption on the RBC structure and function, including morphology, longevity, and gas exchange.

In conclusion, our study provides several key insights into GV interactions with blood components, uncovering mechanisms underlying their recognition by the body and the balance between circulation half-life and contrast enhancement. By understanding the impact of these interactions on performance and safety, we move closer to optimizing GVs as injectable imaging agents and realizing the full potential of this promising technology.

## Supporting Information Available

Methods; Figure S1: dose–response of hemodynamic contrast; Figure S2: ultrasound time courses in NSG mice; Figure S3: modeling of scattering cross section; Figure S4: adsorption in the presence of serum; Figure S5: flow cytometry gating; Figure S6: MALDI-TOF of GVs and discussion of PEG density; Figure S7: impact of CuAAC reagents on hydrostatic collapse pressure; Figure S8: direct functionalization with NHS-PEG; Figure S9: TEM of GVs after serum exposure; Figure S10: ultrasound imaging of GVs in BALB/c serum; Figure S11: peak enhancement following repeated Ana-PEG injection; Figure S12: zwitterionic polymer-coated GV characterization and impact on hemodynamic contrast; Figure S13: scatter plots of protein abundance; Figure S14: heat map of cyanobacterial proteins; Figure S15: protein abundances relative to serum; Figure S16: synthesis of CTA1; Figure S17: synthesis of zwitterionic polymer (PDF)
LC-MS/MS data set (XLSX)
